# Antibiotic-Induced Disruption of Gut Microbiota Alters Local Metabolomes and Immune Responses

**DOI:** 10.3389/fcimb.2019.00099

**Published:** 2019-04-24

**Authors:** Lin Sun, Xiaoyan Zhang, Yuxiao Zhang, Kai Zheng, Qiaoyan Xiang, Ning Chen, Zhiyun Chen, Nan Zhang, Junping Zhu, Qiushui He

**Affiliations:** ^1^Department of Medical Microbiology, Capital Medical University, Beijing, China; ^2^Beijing Pediatric Research Institute, Beijing Children's Hospital, Capital Medical University, National Center for Children's Health, Beijing, China; ^3^Department of Medical Microbiology and Immunology, University of Turku, Turku, Finland

**Keywords:** gut microbiome, microbial metabolite, immune response, cytokine, antibiotic, co-correlation analysis

## Abstract

Gut microbiome plays an essential role in modulating host immune responses. However, little is known about the interaction of microbiota, their metabolites and relevant inflammatory responses in the gut. By treating the mice with three different antibiotics (enrofloxacin, vancomycin, and polymixin B sulfate), we aimed to investigate the effects of different antibiotics exposure on gut microbiota, microbial metabolism, inflammation responses in the gut, and most importantly, pinpoint the underlying interactions between them. Although the administration of different antibiotics can lead to different effects on mouse models, the treatment did not affect the average body weight of the mice. A heavier caecum was observed in vancomycin treated mice. Treatment by these three antibiotics significantly up-regulated gene expression of various cytokines in the colon. Enrofloxacin treated mice seemed to have an increased Th1 response in the colon. However, such a difference was not found in mice treated by vancomycin or polymixin B sulfate. Vancomycin treatment induced significant changes in bacterial composition at phylum and family level and decreased richness and diversity at species level. Enrofloxacin treatment only induced changes in composition at family presenting as an increase in *Prevotellaceae* and *Rikenellaceae* and a decrease in *Bacteroidaceae*. However, no significant difference was observed after polymixin B sulfate treatment. When compared with the control group, significant metabolic shift was found in the enrofloxacin and vancomycin treated group. The metabolic changes mainly occurred in Valine, leucine, and isoleucine biosynthesis pathway and beta-Alanine metabolism in enrofloxacin treated group. For vancomycin treatment metabolic changes were mainly found in beta-Alanine metabolism and Alanine, aspartate and glutamate metabolism pathway. Moreover, modifications observed in the microbiota compositions were correlated with the metabolite concentrations. For example, concentration of pentadecanoic acid was positively correlated with richness of *Rikenellaceae* and *Prevotellaceae* and negatively correlated with *Enterobacteriaceae*. This study suggests that the antibiotic-induced changes in gut microbiota might contribute to the inflammation responses through the alternation of metabolic status, providing a novel insight regarding a complex network that integrates the different interactions between gut microbiota, metabolic functions, and immune responses in host.

## Introduction

The gut microbiome plays an essential role in health and disease of the host. It is well-documented that gut microbiome aids the host in modulating immune responses and protecting against pathogens (Holmes et al., 2011). It also provides beneficial biological functions via production of vitamin and short-chain fatty acids (SCFAs) (Kasubuchi et al., 2015). Disturbing the balance between the host and microbial community can impair these homeostasis and finally result in a series of diseases. Therefore, a better understanding of the mechanistic roles the gut microbiota play in the regulation of host metabolic and immunological functions will provide useful information on the complex host-gut relationship.

Antibiotics have been used as a useful tool to manipulate the gut microbiome, because they cannot only change the structure of host microbial communities but also their function in the gut (Ferrer et al., 2017). Therefore, antibiotics provide a good insight into the potential cause of the microbiota-dependent changes and help us to better understand the host-microbiome crosstalk. In order to explore role of antibiotic on the host's gut microbial function, different antibiotics are used in previous studies, including broad-spectrum or antibiotic cocktails which are targeted at both Gram-negative and Gram-positive bacteria (Rodrigues et al., 2017; Strzepa et al., 2017), mono-antibiosis specific to Gram-positive (Mikkelsen et al., 2015) or Gram-negative bacteria (Oh et al., 2014). In addition, it has also been certificated that antibiotic-induced alternations in gut microbiota are associated with glucose tolerance (Rodrigues et al., 2017), body weight and bone growth (Mikkelsen et al., 2015), and gut microbiome diversity (Nobel et al., 2015). However, there is less attention received on the effects of different kinds of antibiotic usage on the host's gut microbial function.

The antibiotics, usually prescribed for infections, can also target commensal microbiota. Because of their different mechanisms in killing or inhibiting growth of bacteria, antibiotics may have differing effects on composition and richness of gut bacteria. For example, continuous use of therapeutic-dose ampicillin induces microbial dysbiosis and caused enhanced production of NF-κB in the colon tissues of a mouse model (Shi et al., 2018). Antibiotic cocktails which composed of vancomycin, neomycin, ampicillin, and metronidazole can induce huge changes of gut microbiota diversity, and decrease the severity of autoimmune uveitis by increasing Tregs as well as decreasing effector T cells and cytokines in the gut and extraintestinal tissues (Nakamura et al., 2016).

The gut microbiota and related metabolites are critical for host immune response (Kim et al., 2016). However, little is known about interaction of microbiota, their metabolites and relevant inflammatory responses in the gut. Moreover, how microbiota and their metabolites impact the host immunity remains to be explored. Therefore, in the current study, by treating the mice with three different antibiotics including broad-spectrum antibiotic, anti-Gram-positive, and anti- Gram-negative bacteria antibiotic we sought to: (i) study the changes in the gut microbiota induced by different antibiotics, (ii) detect the following metabolic shifts of the gut microbiota, (iii) explore the alternation of inflammation responses, especially the expression of key cytokines in the colon, (iv) identify the similar or specific changes induced by different antibiotics at the above three level, and (v) the most importantly, pinpoint the underlying interactions between microbiota, their metabolites and the host immunity. The results obtained will provide an important basis for clinical relevance of microbiome in human health and disease.

## Materials and Methods

### Ethics Statement

The animal experiments were approved by and conducted in accordance with the guidelines set by the Institutional Animal Care and the animal ethics committees of Capital Medical University.

### Mice and Antibiotics Treatment

**Figure 1:**
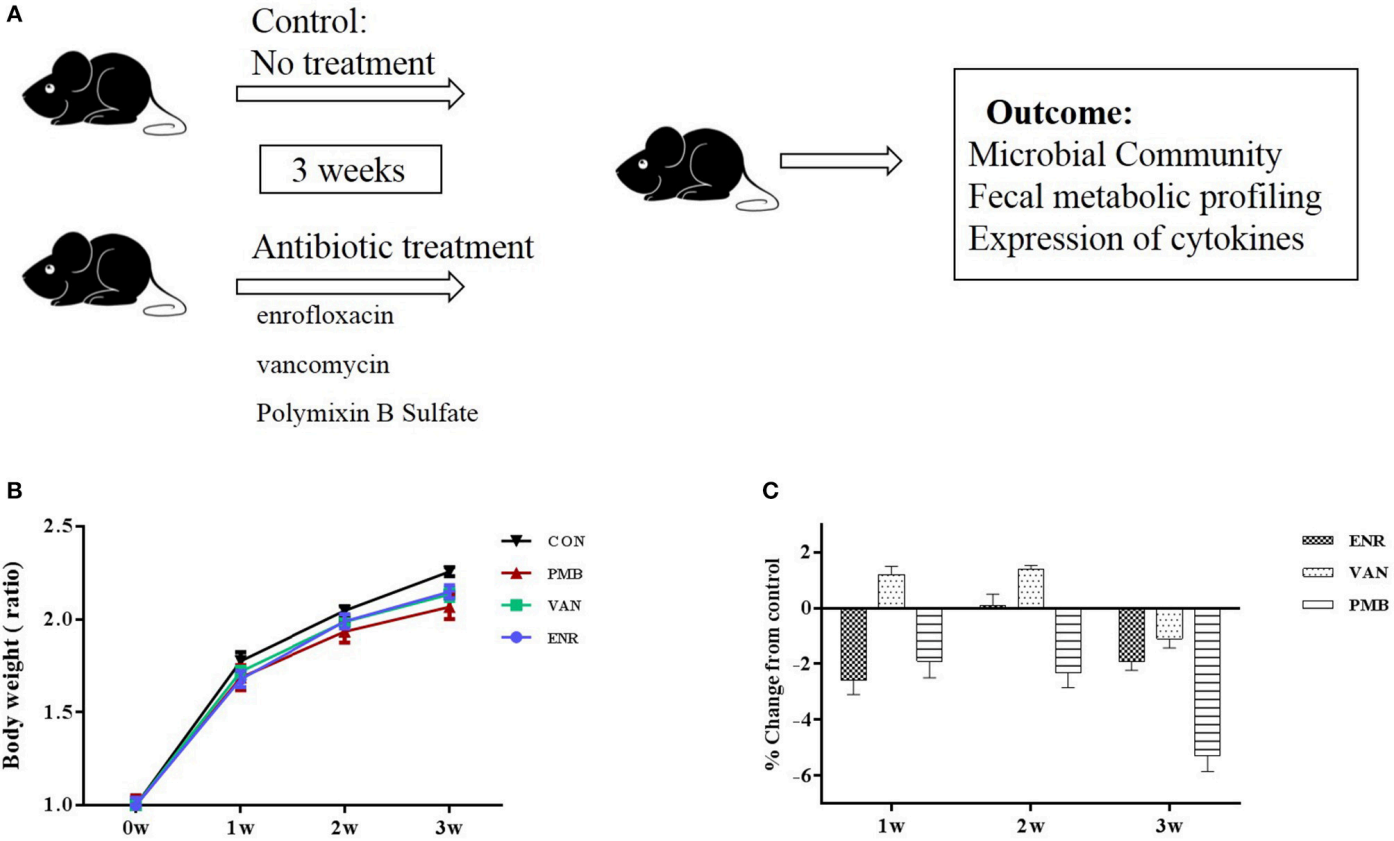
Timeline and body weight results of the experimental procedure. **(A)** Schedule of the experimental procedure in C57BL/6 mice that were treated with different antibiotics. **(B)** Changes in body weight ratio in relation to the respective values at pre-treated time points. **(C)** Growth rates in antibiotic treated groups expressed as percent difference from control. Values are expressed as means ± standard error. ENR, enrofloxacin; VAN, vancomycin; PMB, polymixin B sulfate; CON, control group.

Three-week-old female C57BL/6 mice purchased from Academy of Military Medical Sciences, Beijing, China were housed in the animal facility of the Capital Medical University for 1 week to acclimatize the environment. Mice were randomized into four groups (*n* = 5 per treatment group), and were given single or no antibiotic for 3 weeks to create an altered microbiome. Antibiotics were administered in drinking water in the following concentrations: enrofloxacin (0.27 mg/ml) (Strzepa et al., 2017), vancomycin (0.5 mg/ml), and polymixin B sulfate (0.1 mg/ml) (Oh et al., 2014). Control mice received sterilized water only. Water containers were changed once a day to supply fresh antibiotics. During the treatment, body weight was monitored for each animal every week. The fecal pellets from individual mouse were collected at baseline and 3 weeks. At the end of the experiment (week 3), all mice from four groups were sacrificed and the colons excised. All samples were either fixed in formalin or snap frozen and stored at −80°C until processing. The study design was shown in Figure 1A.

### Colon Hematoxylin-Eosin Staining

Mice were sacrificed on the third weeks after the antibiotic treatment. The distal colon was removed and washed with 0.9% saline. The colon tissue 1 cm proximal to the cecum was cut and then part of it was fixed in 4% formalin and embedded in paraffin for hematoxylin and eosin (HE) staining. The stained sections were observed and photographed under a light microscope (with 200× magnification).

### Cytokine Genes Expression in Colon

The rest parts of the colon tissue were stored dry at −80°C. Total RNA was extracted using the RNAprep Pure Tissue Kit (Tiangen), according to the manufacturer's instructions. Total RNA was quantized by Nanodrop 2,000 (Thermo Fisher Scientific Inc) and 1 μg of total RNA was reverse transcribed to cDNA using EasyScript First-Strand cDNA Synthesis SuperMix (Transgen Biotech). qRT-PCR was performed using TransStart Top Green qPCR SuperMix (Transgen Biotech). The gene of pro-inflammatory cytokines (*IFN-*γ, *TNF-*α, *IL-1*β, and *IL-6*), anti-inflammatory (*IL-4, IL-10*) and effector cytokines of Th17 cell (*IL-17* and *IL-23*) were analyzed. The primers were designed and their sequences were shown in Supplementary Table 1. All results of the target genes were normalized to the housekeeping gene GAPDH as an endogenous control. Reactions were run on an Applied Biosystems 7,500 using the following protocol: 94°C for 30 s, 40 cycles of 5 s at 94°C, 15 s at 58°C, 10 s at 72°C. Results were analyzed using 2^−ΔΔCt^ methods.

### Fecal Bacterial DNA Extraction and 16S rRNA Gene Sequencing

Fecal bacterial DNA from individual mouse was extracted using QIAamp DNA Stool Mini Kit following the manufacturer's instructions (Qiagen). DNA was quantified and 1 ng of the purified DNA was used as template for PCR amplification. The V4 region of 16S rRNA gene was amplified using universal primers (515 F and 806 R). The barcoded amplicons from all samples were normalized, pooled to construct the sequencing library, and then sequenced by an Illumina Miseq to generate pair-ended 250 nt reads.

The raw mate-paired files of 16S rRNA gene sequences were first trimmed to dispose bases with high error probability (>0.01) and merged using FLASH (version 1.2.7). The pre-processed data were then analyzed using QIIME (version 1.9.1) software package (Caporaso et al., 2010). Operational taxonomic units (OTUs) were clustered with 97% sequence similarity using UPARSE (version 7.0.1001). The normalized OUT tables were used for diversity and statistical analyses. Biodiversity of the samples (alpha-diversity) was calculated with Chao 1 and Shannon indexes. Similarities between samples (beta-diversity) were calculated by weighted UniFrac. Curtis similarity clustering analysis was used to perform a principal coordinate analysis (PCoA).

### Metabolomics Analyses

Metabolic compounds in fecal samples were isolated using the previously described method with minor modifications (Ng et al., 2012). Briefly, 40 mg of feces were vortexed with 1 ml of cold methanol for 1 min, followed by ultrasound treatment in ice water for 10 min. The supernatants were transferred to a high-performance liquid chromatography (HPLC) vials and dried for 2 h in a SpeedVac, followed by derivatization with methoxyamine-HCl and N,O-bis(trimethylsilyl)trifluoroacetamide (BSTFA). After incubated at 37°C for 90 min, the derivatized samples were centrifuged at 12,000 rpm for 10 min. Then, the supernatants were injected (1 μl) into an Agilent GC-MS that was running in full scan mode. The injector was set at 280°C, transfer line at 150°C and the ion source at 230°C. The initial oven temperature was set to 60°C, increasing at 60°C/min for 2 min, and subsequently increasing at a rate of 10°C/min to 300°C. The mass spectrometer was set to scan the range *m*/*z* 35–750.

XCMS was used for peak-picking, alignment and extraction of the peak intensities. Univariate statistical analysis was conducted using SPSS v.17 where a paired *t*-test was performed to compare metabolite intensities between the infected and non-infected groups and *p* ≤ 0.05 was considered statistically significant. Partial least squares discriminant analysis (PLS-DA) was used to compare the metabolomics profiles between the control and lead-treated groups. Molecular features with significant changes (*p* < 0.05 and fold change > 1.5 or <0.667) were identified using the National Institute of Standards and Technology (NIST) Standard Reference Database and searched against the KEGG Database to obtain pathway information.

### Statistical Analysis

Statistical analysis was carried out with tests using the SPSS software package. A two-tailed Student's *t*-test and a non-parametric Kruskal-Wallis test was used, respectively, to compare the normal distribution or abnormal distribution data between the controls and the antibiotic treated mice. Spearman's rank correlation coefficients (two-tailed test) were calculated for co-occurrence analysis. A *p*-value of 0.05 or less was considered statistically significant.

## Results

### Effect of Antibiotic Treatment on Growth Performance and Gut Phenotype

**Figure 2:**
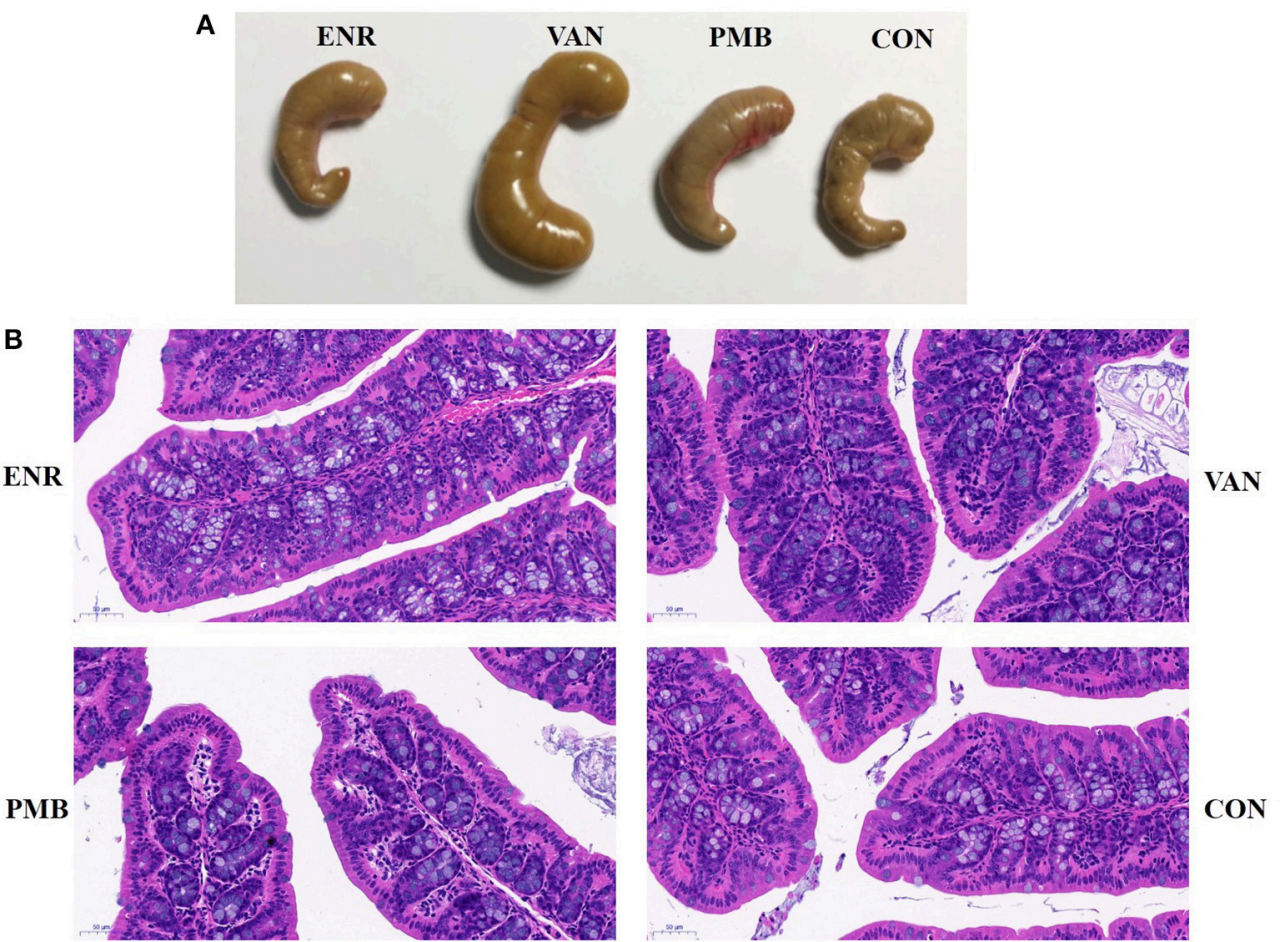
Effect of antibiotic treatment on caecal volume and histological sections of the colon. **(A)** The caecal volume of mice after treated with antibiotics for 3 weeks. **(B)** Pathology of colon tissues (HE stain, ×200). ENR, enrofloxacin; VAN, vancomycin; PMB, polymixin B sulfate; CON, control group.

Antibiotic treatment did not affect the average body weight of the mice, when compared with control mice (all *p* > 0.05 at each time point (Figures 1B,C). At 3 weeks, vancomycin treated mice exhibited a pronounced increase in caecal volume (Figure 2A). The histopathological results showed that no structure change of the colon was found in the antibiotic treated mice (Figure 2B).

### Effect of Antibiotics on the Inflammation Mediators in Mouse Colon

**Figure 3:**
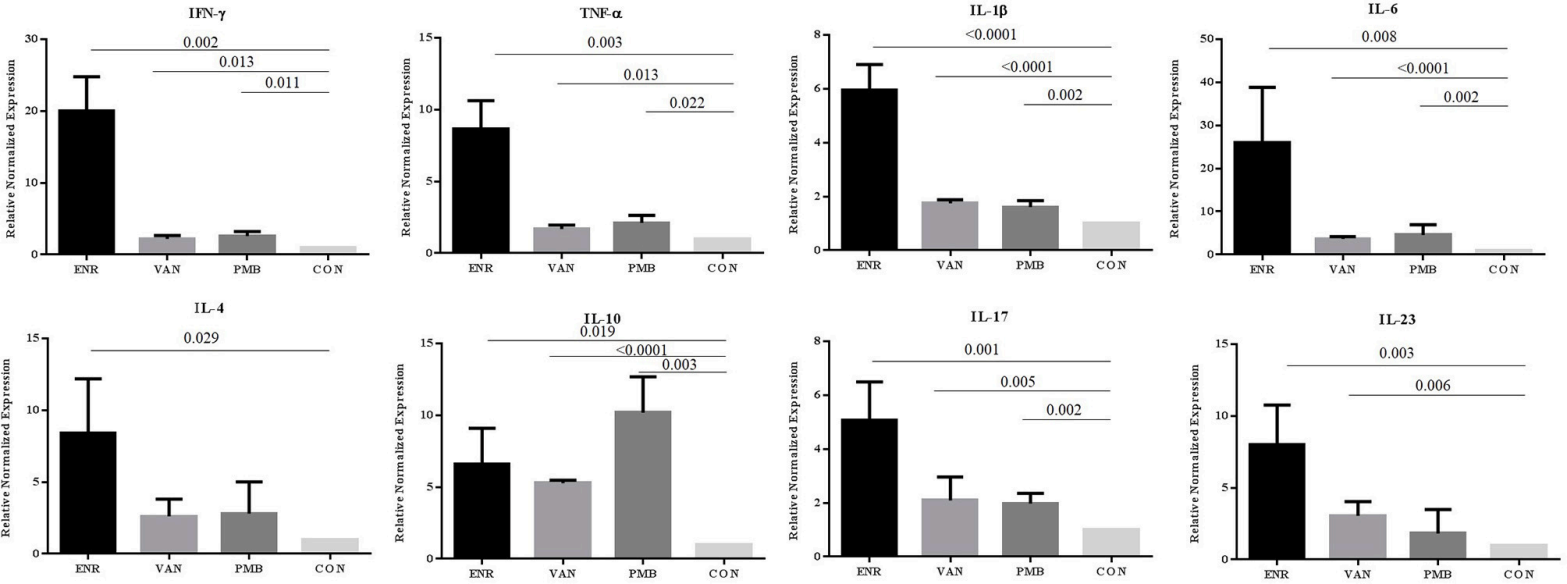
Changes in gene expression of different inflammatory cytokines determined at mRNA level in colon. ENR, enrofloxacin; VAN, vancomycin; PMB, polymixin B sulfate; CON, control group.

To explore the effect of gut microbiota on the gut inflammation in mice, we analyzed the expression of inflammation associated cytokines on mRNA level (Figure 3). Notably, all three antibiotics treatment significantly up-regulated the gene expression of pro-inflammatory *IFN-*γ, *TNF-*α, *IL-1*β, and *IL-6* (all *p* < 0.05), especially after the enrofloxacin treatment. Similar trends were found in the anti-inflammatory *IL-4* and effector cytokines of Th17 cell subset *IL-17* and *IL-23* (*P* < 0.05). The gene expression of anti-inflammatory *IL-10* were also up-regulated in the three antibiotics treatment group (*P* < 0.05), while the polymixin B sulfate seemed to have a stronger affection. When the expression of *IFN-*γ*/IL-4* was compared the ratio turned to be higher in enrofloxacin treated group than control group (2.39 vs. 1.00), which suggested an increased response of Th1 cell than Th2. However, no such difference was found in groups with the treatment by vancomycin and polymixin B sulfate (Supplementary Figure 1).

### Microbial Alterations After the Treatment of Antibiotics

**Figure 4:**
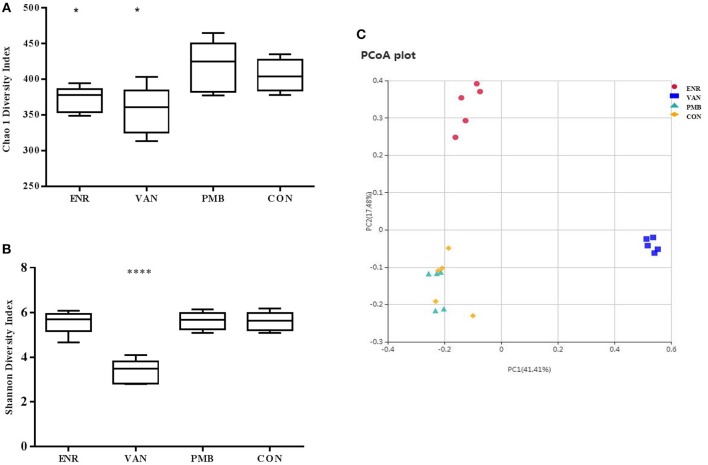
Effect of antibiotic treatment on diversity of gut microbiota composition. **(A,B)** Chao 1 and Shannon indexes of fecal samples in control and three antibiotic treatment groups. **(C)** Principal coordinate analysis of fecal samples in control and three antibiotic treatment groups by curtis similarity clustering analysis. *p < 0.05 compared to the control and PMB groups; ****p < 0.0001 compared to other three groups. ENR, enrofloxacin; VAN, vancomycin; PMB, polymixin B sulfate; CON, control group.

Microbiota composition was surveyed by sequencing the 16S rRNA gene from the fecal pellets. A total of 1,750,381 V4 16S rRNA sequence reads from 20 samples after antibiotics treatment, with an average 87,519 (ranging from 69,542 to 99,694 reads per sample) sequence reads per sample were used for subsequent analysis. The gut microbiome of the mice in different groups were similar before the antibiotics treatment (Supplementary Figure 2). However, vancomycin decreased the species richness and diversity indices compared to that in the control group, as reflected by the Chao 1 and Shannon index with statistical differences. A decreased species richness of gut microbiota was detected in mice treated with enrofloxacin. However, as compared with the controls, polymixin B sulfate did not affect both species richness and diversity indices (Figures 4A,B). The PCoA result indicated vancomycin and enrofloxacin treated mice showed a district separation in microbiota composition from both polymixin B sulfate treated group and control group (Figure 4C).

**Figure 5:**
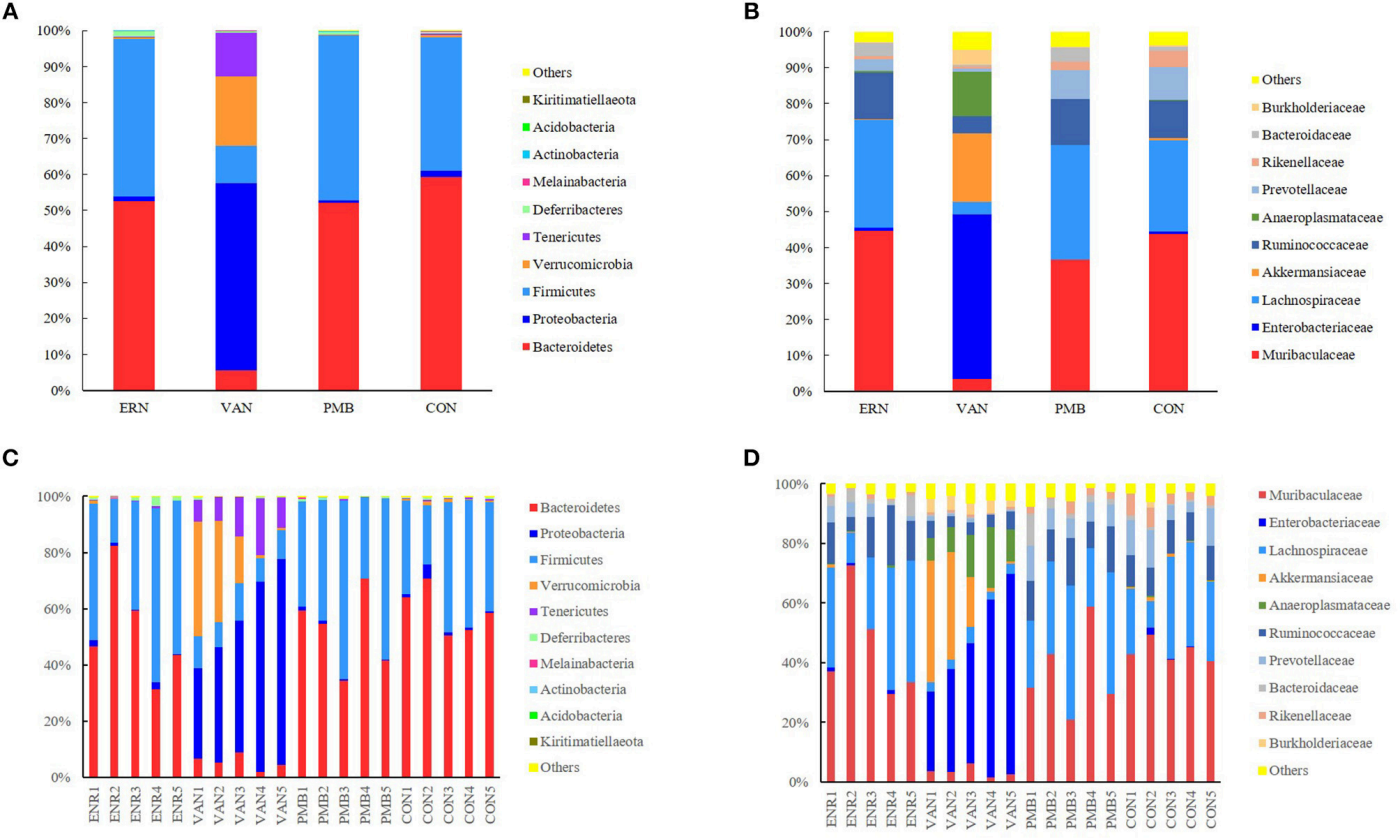
Taxonomic plots showing relative abundance across the different groups after 3 weeks treatment. **(A)** The average phylum-level taxonomic composition in each group. **(B)** The average family-level taxonomic composition in each group. **(C)** The phylum-level taxonomic composition in each mouse. **(D)** The family-level taxonomic composition in each mouse. ENR, enrofloxacin; VAN, vancomycin; PMB, polymixin B sulfate; CON, control group.

At phylum level, the *Bacteroidetes* and *Firmicutes* were the most predominant phylum in the mice without antibiotic treatment, accounting for 59.3 and 37.1% of total sequences, respectively. After 3 weeks treatment by vancomycin, when compared with control group, a significantly increased abundance of bacteria belonging to the phyla *Proteobacteria* (52.1 vs. 1.7%, *P* = 0.0002) as well as the *Tenericutes* phylum (12.2 vs. 0.4%, *P* = 0.0009) were detected, while a concomitant decrease of *Bacteroidetes* (5.5 vs. 59.3%, *P* < 0.0001), *Firmicutes* (10.4 vs. 37.1%, *P* = 0.0009), *Melainabacteria* (0.06 vs. 0.2%, *P* = 0.03) was also detected. Treatment by enrofloxacin and polymixin B sulfate did not produce detectable modification in the composition of the fecal microbiota at phylum level, and no significant differences of bacterial abundance were found between the treated groups and the control group (all *P* > 0.05) (Figures 5A,C).

When the analysis was performed at lower taxonomic ranks, a more significant changes were identified in some other taxa. At family level, following 3 weeks vancomycin intervention, an increase of bacterial abundance was identified in the *Enterobacteriaceae* (45.7 vs. 0.6%, *P* < 0.0001), *Akkermansiaceae* (19.1 vs. 0.7%, *P* = 0.06), *Anaeroplasmataceae* (12.2 vs. 0.2%, *P* = 0.0008), *Burkholderiaceae* (4.1 vs. 0.6%, *P* = 0.0004), and a decrease of abundance in *Muribaculaceae* (3.5 vs. 43.8%, *P* = 0.0001), *Lachnospiraceae* (3.6 vs. 25.3%, *P* = 0.002), *Ruminococcaceae* (4.8 vs. 10.4%, *P* = 0.0001), *Prevotellaceae*(0.9 vs. 9.1%, *P* = 0.004), *Rikenellaceae* (0.7 vs. 4.6%, *P* = 0.003), and *Bacteroidaceae* (0.3 vs. 1.0%, *P* = 0.01). Relatively, a weaker modification was induced by enrofloxacin in three family members with low abundance, including *Prevotellaceae* (3.4 vs. 9.1%, *P* = 0.04), *Rikenellaceae* (0.9 vs. 4.6%, *P* = 0.005), and *Bacteroidaceae* (3.6 vs. 1.0%, *P* = 0.04). However, no significant differences in bacterial abundance was found between the polymixin B sulfate treated groups and the control group (*P* > 0.05) (Figures 5B,D). A significantly change of bacterial abundance were also detected in vancomycin treated group at genus level (Supplementary Figure 3).

### Metabolic Alterations After the Treatment of Antibiotics

**Figure 6:**
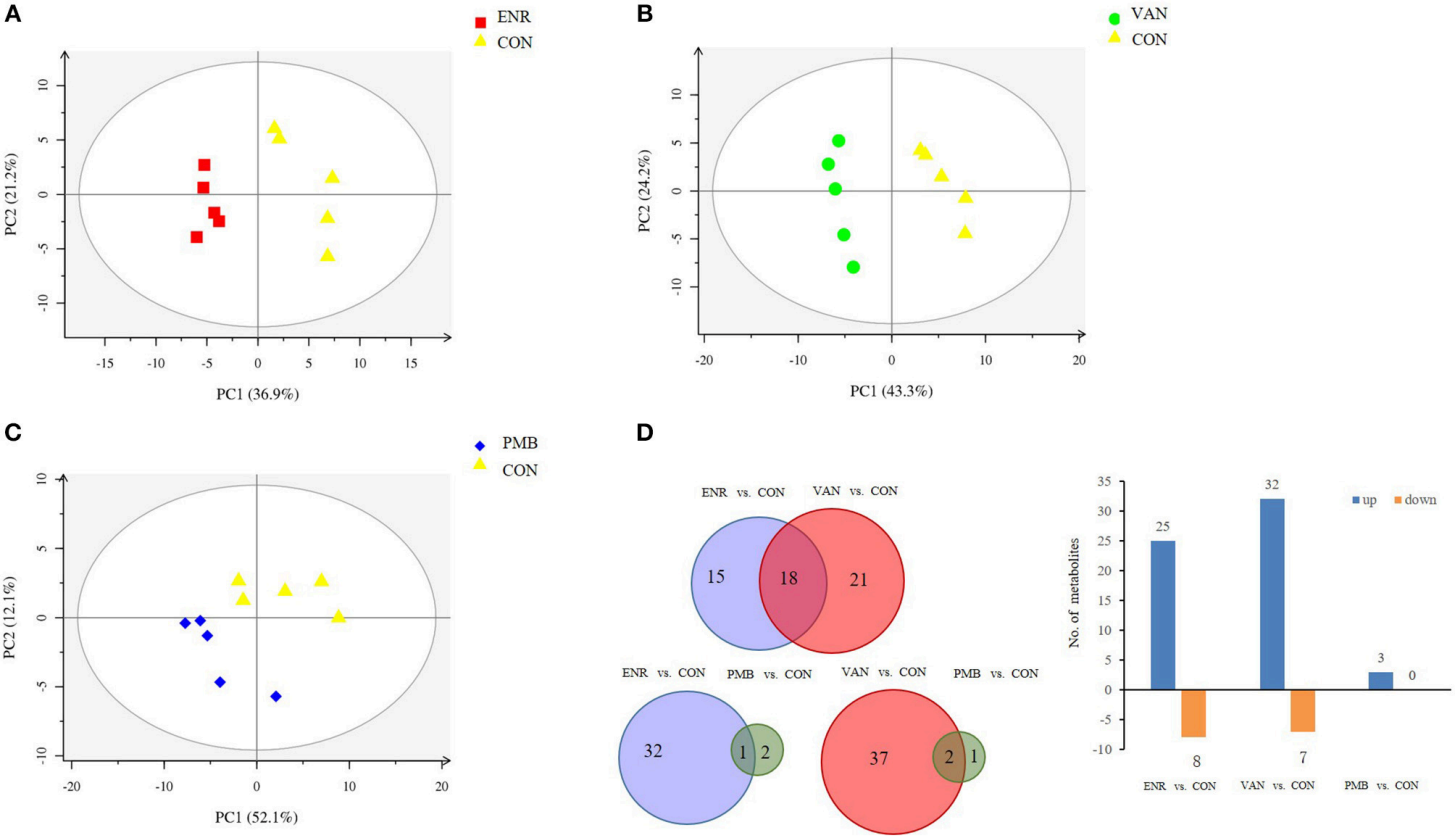
Metabolomics profile of antibiotics intervention on mice. **(A–C)** The PLS-DA score plots of mice fecal samples, respectively, for enrofloxacin (ENR), vancomycin (VAN), and polymixin B sulfate (PMB). **(D)** Different metabolites selected through GC-MS analysis.

**Figure 7:**
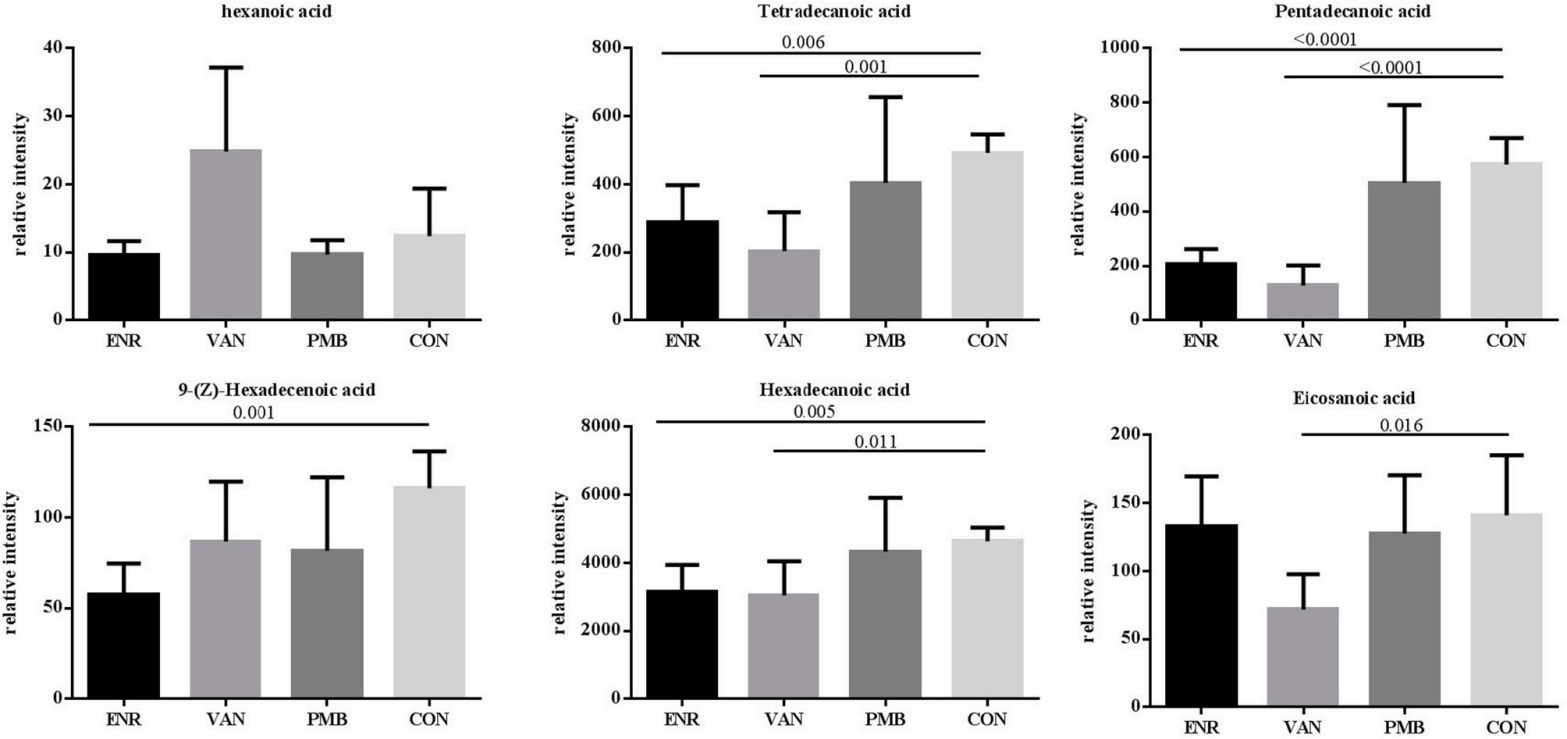
Effects of antibiotics on the microbial metabolites in fecal samples of mice. Values are expressed as means ± standard error. ENR, enrofloxacin; VAN, vancomycin; PMB, polymixin B sulfate; CON, control group.

Fatty acid and other metabolites were investigated using untargeted GC-MS analysis. When compared with the control group, significant separation trend was observed in the vancomycin treated group (*R*^2^ = 0.86, *Q*^2^ = 0.48) as well as the enrofloxacin treated group (*R*^2^ = 0.95, *Q*^2^ = 0.75). Relatively, a weaker separation trend was observed between the polymixin B sulfate treated group and the control group (*R*^2^ = 0.86, *Q*^2^ = 0.2) (Figures 6A–C). As is shown in Figure 6D, a total of 54 discriminative metabolites were detected between the antibiotics treatment groups and the control group, and 39 of them were upregulated. The detailed information of the identified metabolites were shown in Supplementary Table 2. Among them, five metabolites were classified as long-chain fatty acid (LCFA). In addition, a higher level of hexanoic acid belonging to SCFA was observed in vancomycin treated group when compared with the controls (24.82 ± 5.51 vs. 12.37 ± 3.14, *P* = 0.08) (Figure 7).

**Figure 8:**
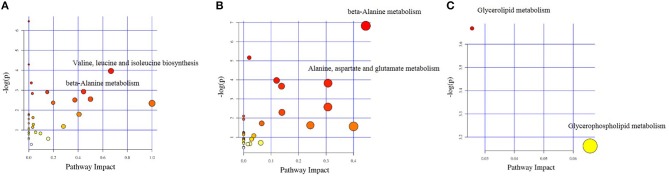
Impact factors of pathways calculated by KEGG pathway analysis. The major metabolic pathways in which the altered metabolites between antibiotic treated group and control group enriched were shown based on the *p-*value and impact factor. **(A)** The major metabolic pathways in which the altered metabolites between enrofloxacin (ENR) group and control group enriched. **(B)** The major metabolic pathways in which the altered metabolites between vancomycin (VAN) group and control group enriched. **(C)** The major metabolic pathways in which the altered metabolites between polymixin B sulfate (PMB) group and control group enriched.

The different chemical metabolites were further performed with pathway analysis using the KEGG PATHWAY Database. Using a *p*-value cut-off of <0.05 and an impact factor threshold of >0, we noticed that enrofloxacin induced metabolic changes were mainly enriched in Valine, leucine, and isoleucine biosynthesis metabolism pathway and beta-Alanine metabolism, with impact factors of 0.67 and 0.44, respectively (Figure 8A). Vancomycin induced metabolic changes were mainly enriched in beta-Alanine metabolism and Alanine, aspartate, and glutamate metabolism pathway, with impact factors of 0.44 and 0.31, respectively (Figure 8B). The 3 discriminative metabolites detected in polymixin B sulfate treated group were mainly enriched in Glycerolipid metabolism and Glycerophospholipid metabolism pathway, with impact factors of 0.03 and 0.07, respectively (Figure 8C).

### Correlations Between Intestinal Microbiome and Metabolome

**Figure 9:**
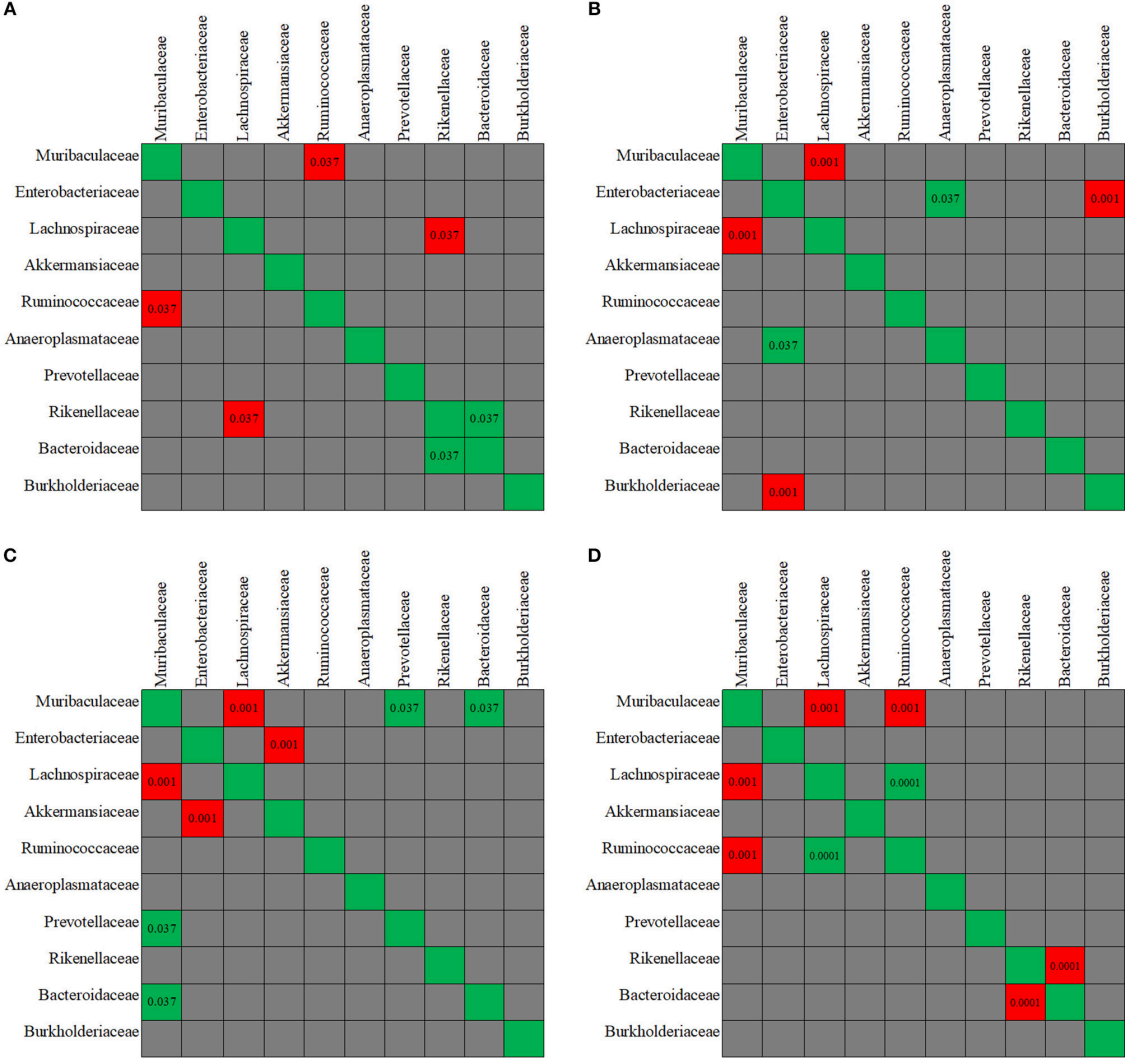
The co-variance between the fecal microbiota members. Individual *P*-value was shown inside each square, and only significant difference was indicated. Red color of *P*-value indicates negative correlation, whereas green color positive correlation. **(A-D)** The co-variance between the fecal microbiota members, respectively, for in control, enrofloxacin, vancomycin, and polymixin B sulfate group.

The evaluation of taxa coexistence indicated that the co-occurrence/co-exclusion of microbial taxa existed in the gut microbiome of the control mice. As shown in Figure 9A, a negative correlation was present in control group between the family *Muribaculaceae* and *Ruminococcaceae*, and between *Rikenellaceae* and *Lachnospiraceae* (all *p* < 0.05). *Rikenellaceae* showed a positive correlation with *Bacteroidaceae* family. However, after the mice were treated with antibiotics, the correlation observed above was disturbed. The changes in the co-occurrence/co-exclusion of microbial taxa differed in the three antibiotic groups (Figures 9B–D).

**Figure 10:**
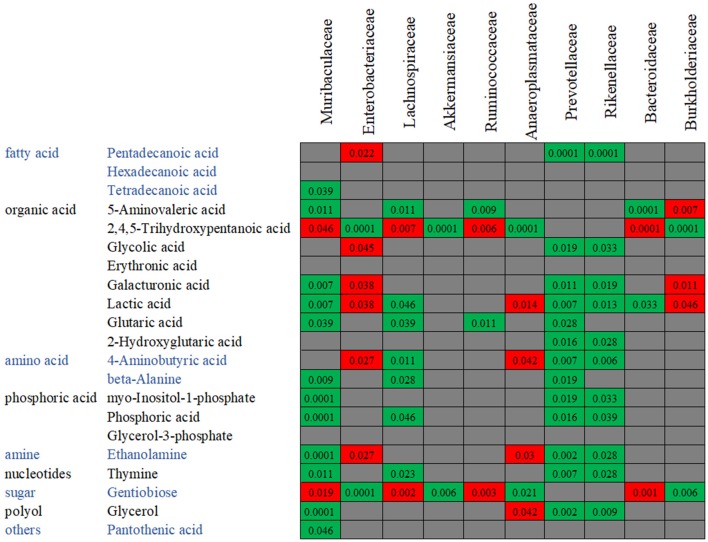
The co-variance between the bacterial species abundance and the abundance of metabolites. Individual *P*-value was shown inside each square, and only significant difference was indicated. Red color of *P*-value indicates negative correlation, whereas green color positive correlation.

In order to confirm the relationships between the gut microbiota composition and the metabolite concentration, a co-correlation analysis was performed (Figure 10). Among the 21 overlapping metabolites which were identified in the antibiotics-treated groups, most of the discriminative metabolites are associated with the microbiota abundances. For example, as reported above, the abundances of *Rikenellaceae* and *Prevotellaceae* were decreased by both enrofloxacin and vancomycin, and consequently, reductions in these families may in turn cause 13 metabolites to low productions. We also found one LCFA, pentadecanoic acid was negatively correlated with *Enterobacteriaceae* while positively correlated with *Rikenellaceae* and *Prevotellaceae*.

## Discussions

Gut bacterial colonization at birth and the evolution and development after birth are critical in priming the proper development of the immune system of the host. Gut microbiota continually provide appropriate signals to immune cells which helps to maintain a steady immunological state and serve as a barrier against pathogens (Zárate-Bladés et al., 2016). It is well-described that the diversity, functional capacity and age-associated dynamics of the gut microbiome are associated with diseases ranging from localized gastroenterologic disorders to systemic diseases, such as autoimmune diseases (Vatanen et al., 2016) and diabetes (Qin et al., 2012). However, very few effort is currently concentrated on exploring the underlying mechanisms of alteration of gut microbiome on gut bacterial metabolome. Therefore, in this study, we established antibiotic treated mouse models and performed the exploratory study on how these antibiotics modified the intestinal microbial community and metabolism and their effect on the host immune status and gut barrier function.

Previous studies observed that antibiotic induced perturbation of the microbiota was accompanied by modulation of inflammatory state of the host. For example, mice with dysbiosis induced by therapeutic-dose ampicillin exhibited an increased production of IFN-γ and a decrease in secretory IgA in the colon (Shi et al., 2018). The modifications of the microbiota upon vancomycin treatment could greatly impact immune cells of lamina propria (Candon et al., 2015). Consistently, we found in the present study that after the treatment by enrofloxacin, vancomycin, or Polymixin B sulfate all mice experienced an alternation in cytokine expression in the colon. But the degree and type of changes differed among the three treatments. Enrofloxacin treated mice seemed to have an increased Th1 response in the colon. However, such a difference was not observed in mice treated by vancomycin or polymixin B sulfate. However, these different antibiotic treatments did not affect animal body weight, suggesting that the observed modulation of immunological response is not a result of side effects related to antibiotic treatment. To explore the underlying mechanisms of the altered immunological responses in the gut, the microbiota composition and the metabolism were further analyzed.

In this study, three kinds of antibiotics were used to build the mice models, including the broad-spectrum antibiotic enrofloxacin, gram-positive bactericidal antibiotic vancomycin, and gram-negative bactericidal antibiotic polymixin B sulfate. These antibiotics lead to different effect on mice. According to our results of the 16S results, a decreased species richness of gut microbiota, decreased abundances of *Prevotellaceae* and *Rikenellaceae*, and an increased abundance of *Bacteroidaceae* were detected in mice treated with enrofloxacin. Consistent with previous studies, vancomycin treatment induced significant changes in bacterial composition and richness. The relative abundances of the top ten families with highest abundance were all changed after the treatment of 3 weeks. However, no significant difference was detected among the families between the polymixin B sulfate treated and water treated group. Consistent with the finding in bacterial composition and richness, the microbiota metabolic status in mice treated by enrofloxacin or vancomycin was significantly distinguished from the control mice, whereas most of the metabolites detected in mice treated by polymixin B sulfate was similar to the control group. As for the expression profiles of cytokines, a significantly stronger Th1 response was only observed in mice treated by enrofloxacin, but not in mice treated by vancomycin. However, the Th1/Th2 balance as shown by expression profiles of cytokines stayed stable in mice treated by polymixin B sulfate.

Some of the changes found in our studies were consistent with previous studies. After treated with enrofloxacin, significant alteration in cytokine expressions were detected, including higher levels of IFN-γ and IL-17A in arthritis model mice (Dorozynska et al., 2014) and higher TNF-α, IL-1β levels in chicken models (Wisselink et al., 2017). Vancomycin-treated mice have experienced an altered profiles of inflammatory cytokines in the ileum and liver (Jena et al., 2017), and increased Treg cell levels on the ileum and colon lamina propria (Candon et al., 2015). Similarly, a heavier caecum and a dramatic change in the bacterial community was found in feces of mice treated with vancomycin (Tulstrup et al., 2015). Remarkably, the anaerobic bacterial load was increased, with lower diversities and species richness, characterized by a decrease in *Firmicutes* phylum and an increase in *Proteobacteria* (Tulstrup et al., 2015). In a polymixin B treated mouse model, the hepatic and ileal expression of gene *IL-1*β and *IL-6* were decreased (Jena et al., 2017).

Based on the results from our study and previous reported studies, we can get a conclusion that the differences in composition and function of gut microbial communities may contribute to inter-individual variation in cytokine responses to microbial stimulations in host. The components of the gut bacteria interacted with the mucosal and systemic immune systems through specific pathway and cytokine. For example, one study detected a microbiome-cytokine interaction patterns between *Odoribacter splanchnicus* and the genus *Bilophila* and TNF-α production (Schirmer et al., 2016). In addition, bacterial recognition is dependent on transmembrane pattern recognition receptors, including the structurally homologous toll-like receptors (TLR) and intracellular NOD-like receptor (NLR) family. The interaction between IL-10 and TLR2 played an important role in maintaining normal epithelial cell homeostasis in the interplay with commensal enteric bacteria (Ruiz et al., 2006).

One mechanism underlying the microbiome-cytokine interaction may be mediated by the metabolites originated from gut microbiota. It has been reported that the microbial metabolic processes had strong impact on cytokine production (Moffett and Namboodiri, 2003; Nowak et al., 2012). Schirmer et al. validated that production of TNF-α and IFN-γ in blood is associated with specific microbial metabolic pathways, including palmitoleic acid metabolism and tryptophan degradation to tryptophol (Schirmer et al., 2016). It is known that fatty acid also plays an important role in host immunity, including support epithelial cell integrity, innate immune functions, Schauber et al. (2003). In this study, production of three LCFAs including pentadecanoic acid, hexadecanoic acid, and tetradecanoic acid was significantly decreased in antibiotic treated groups. Among them, pentadecanoic acid and tetradecanoic acid were associated with the abundance of gut microbiota. Generally, LCFAs detected in the gut are derived from either lipolysis of dietary fats or host/bacterial fatty acid synthesis (Niot et al., 2009). Several odd-numbered carbon LCFA such as pentadecanoic acid and heptadecanoic acid can be produced by bacteria only (Kumagai et al., 2012). It seems that hyperactive bacterial LCFA synthesis and the high level of intraluminal LCFA was contributed from the increased abundance of genera *Prevotella, Lactobacillus*, and *Alistipes* (Zhao et al., 2018). In our study, pentadecanoic acid is positively correlated with family *Prevotellaceae* and *Rikenellaceae*. Moreover, the mice treated with enrofloxacin presented with decreased abundance of *Prevotellaceae* and *Rikenellaceae*, decreased concentration of pentadecanoic acids and the increased production of TNF-α. This finding is in line with the result reported by Debierre-Grockiego et al. in which four LCFAs including pentadecanoic, myristic, palmitic, and palmitoleic acids could inhibit glycosylphosphatidylinositol-induced TNF-α production by macrophages (Debierre-Grockiego et al., 2006). However, we did not find such correlations in concentrations of myristic, palmitic, or palmitoleic acids with production of TNF-α in our study, suggesting that these four LCFAs may not have the same function in the animal model and further verification is needed.

The results obtained in this study support the hypothesis that the antibiotic-induced alterations in gut microbiota may contribute to the inflammation responses through the alternations of metabolic status. However, it should be kept in mind that the gut microbiome presented with the co-occurrence/co-exclusion function as a network.

There were some limitations in the study. First, the number of mice included in each group was limited, therefore the results should be interpreted with caution. Secondly, the mice used in this study were 4 weeks old and not adult mice, therefore there could be some individual variations in microbiome studied between mice. However, the average relative abundance across different groups of mice before the antibiotics treatment was similar at phylum-level. Thirdly, Alternation of the gut microbiota composition and function is dependent on the classes and doses of antibiotic used as well as route and duration of antibiotic administration (Lange et al., 2016). In this study we have used oral administration of antibiotics for a period of 3 weeks. Follow up analysis was not performed to test recovery of the altered microbiota. Fourthly, the underlying mechanism of the host-microbiota interaction is not explored in the present study. Further studies are needed to uncover the mechanism(s).

In conclusion, our study indicated that 3 weeks antibiotic administration induced elevated expression of many cytokines in mouse colon, suggesting that there might be a direct interaction between antibiotics and host tissue. Furthermore, different antibiotics used can lead to different effects on mouse models. Broad spectrum antibiotic enrofloxacin had a stronger effect on the cytokines response in the colon. Vancomycin can induce significant changes in composition and metabolic profiling of gut microbiota. Compared to enrofloxacin and vancomycin, polymixin B sulfate had a weakest effect. The differences observed in composition and function of gut microbial communities may contribute to inter-individual variation in cytokine responses to microbial stimulations in host. Our study provides a novel insight regarding a complex network that integrates the different interactions between gut microbiota, metabolic functions, and host immunity.

## Ethics Statement

The animal experiments were approved by and conducted in accordance with the guidelines set by the Institutional Animal Care and the animal ethics committees of Capital Medical University.

## Author Contributions

LS and QH conceived and designed the study. LS, XZ, YZ, KZ, QX, NC, ZC, NZ, and JZ performed the experiments. LS and QH analyzed data and wrote the manuscript. All authors reviewed and approved the final manuscript.

### Conflict of Interest Statement

The authors declare that the research was conducted in the absence of any commercial or financial relationships that could be construed as a potential conflict of interest.
