# Supplementary figures and images for: Antibiotic-Induced Disruption of Gut Microbiota Alters Local Metabolomes and Immune Responses
Source: Front Cell Infect Microbiol. 2019 Apr 24;9:99. doi: 10.3389/fcimb.2019.00099 (PMC6491449; doi:10.3389/fcimb.2019.00099)

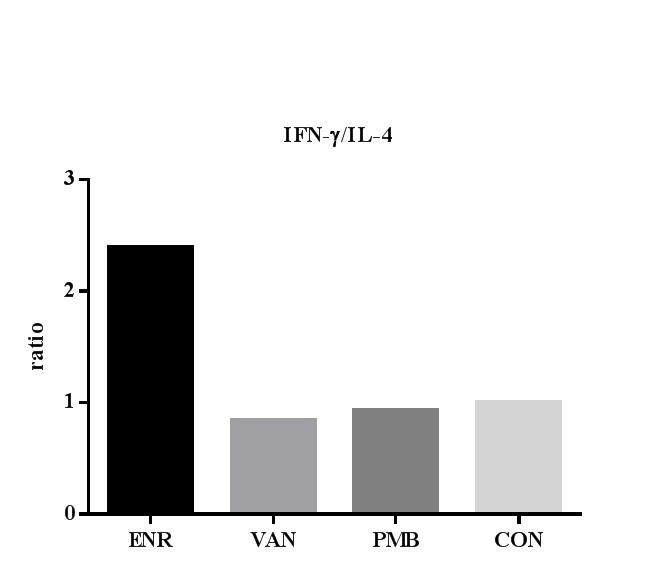

Supplement: Supplementary file 1 [file Image_1.JPEG]

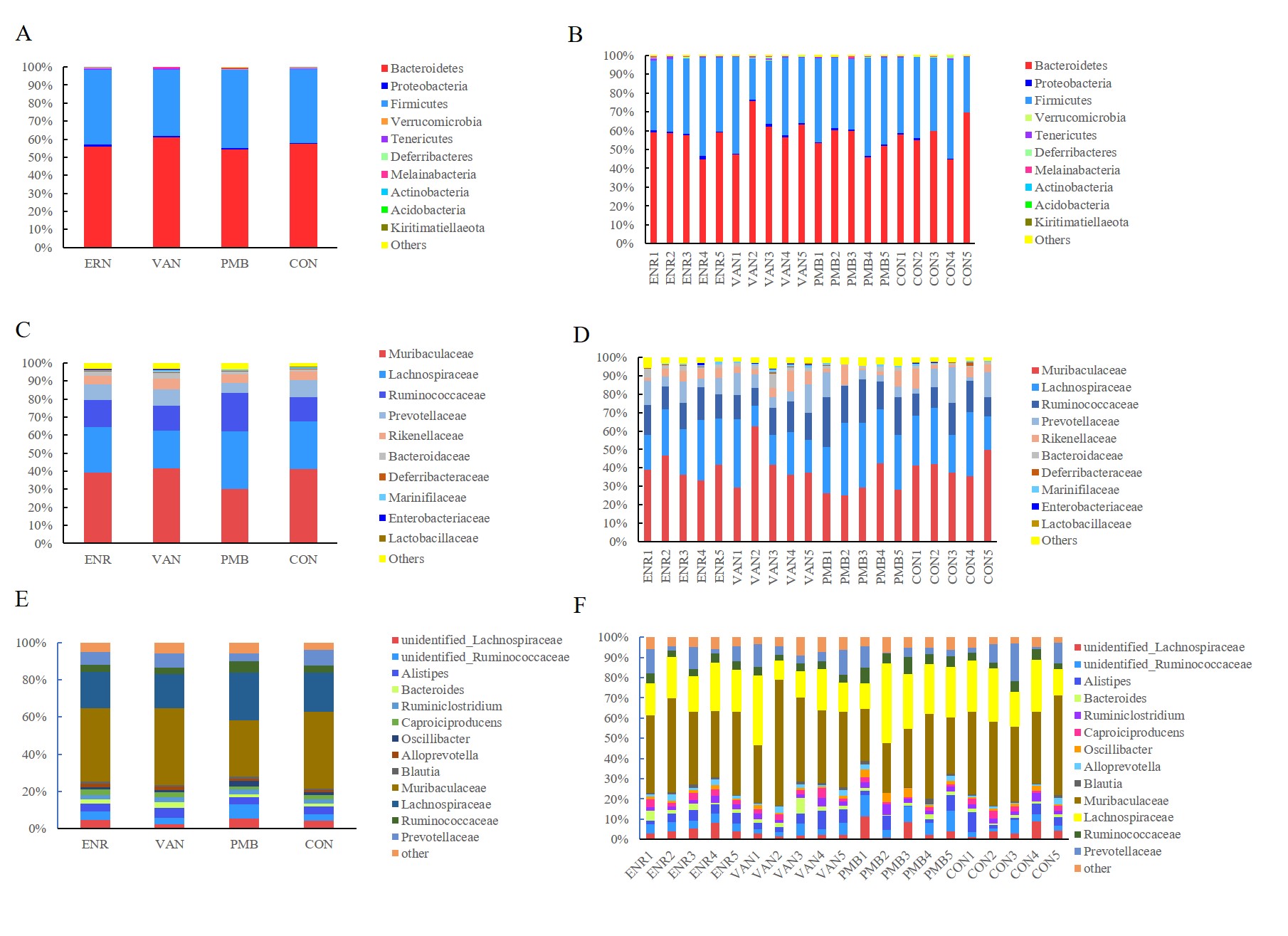

Supplement: Supplementary file 2 [file Image_2.JPEG]

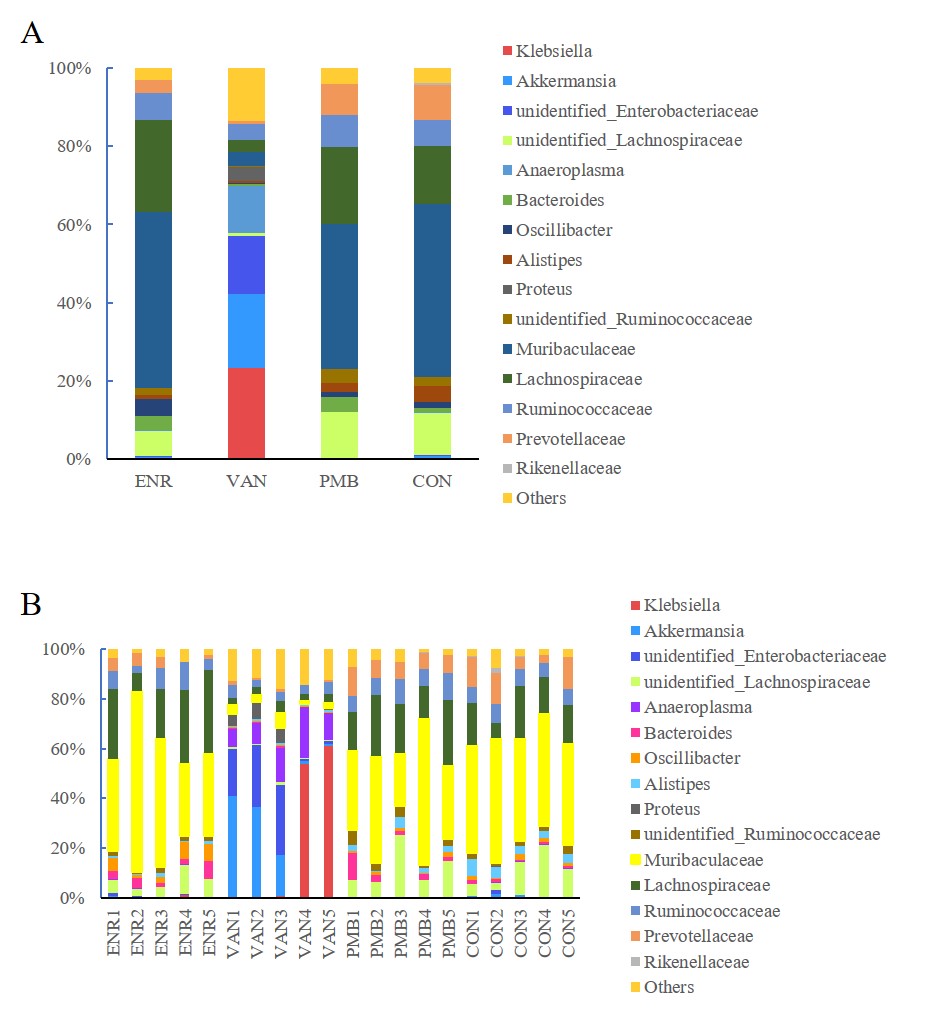

Supplement: Supplementary file 3 [file Image_3.JPEG]
