# Supplementary material for: Antibiotic-Induced Disruption of Gut Microbiota Alters Local Metabolomes and Immune Responses
Source: Front Cell Infect Microbiol. 2019 Apr 24;9:99. doi: 10.3389/fcimb.2019.00099 (PMC6491449; doi:10.3389/fcimb.2019.00099)
Supplement: Supplementary file 4 [file Table_1.DOCX]

Legends to figures

**Supplementary Figure 1.** The ratio of *IFN-γ* and *IL-4* gene expression on mRNA level in colon. ENR., enrofloxacin; VAN., vancomycin; PMB., polymixin B sulfate; CON., control group.

**Supplementary Figure 2.** Taxonomic plots showing average relative abundance across the different groups and in each individual mouse before antibiotics treatment at phylum-level. (A-B) phylum-level. (C-D) family-level. (E-F) genus level.

**Supplementary Figure 3.** Taxonomic plots showing average relative abundance across the different groups and in each individual mouse after 3 weeks antibiotics treatment at genus-level.

**Supplementary Table 1.** Primers’ sequences of cytokine gene used for qRT-PCR

| Cytokine Gene | Primers’ sequences | Length(bp) |
| --- | --- | --- |
| *IFN-γ* | F:5’-TCAAGTGGCATAGATGTGGAAGAA-3’  R: 5’-TGGCTTGCAGGATTTTCATG-3’ | 92 |
| *TNF-α* | F: 5’- CCCTCACACTCAGATCATCTTCT-3’  R: 5’- GCTACGACGTGGGCTACAG-3’ | 61 |
| *IL-1β* | F: 5’- GCAACTGTTCCTGAACTCAACT-3’  R: 5’- ATCTTTTGGGGTCCGTCAACT-3’ | 89 |
| *IL-6* | F: 5’- TAGTCCTTCCTACCCCAATTTCC-3’  R: 5’- TGGTCCTTAGCCACTCCTTC-3’ | 75 |
| *IL-4* | F: 5’- ACAGGAGAAGGGACGCCAT-3’  R: 5’- GAAGCCCTACAGACGAGCTCA-3’ | 95 |
| *IL-10* | F: 5’- ATAACTGCACCCACTTCCCA-3’  R: 5’- TCATTTCCGATAAGGCTTGG-3’ | 177 |
| *IL-17* | F: 5’- CTCCAGAAGGCCCTCAGACTAC-3’  R: 5’- GGGTCTTCATTGCGGTGG-3’ | 169 |
| *IL-23* | F: 5’- GAGGTGGACTGGACTACCGA-3’  R: 5’- GGAACTGCTACTGCTCTTGA-3’ | 318 |
| *GAPDH* | F: 5’-CATCACTGCCACCCAGAAGA-3’  R: 5’-TGAAGTCGCAGGAGACAACC-3’ | 320 |

**Supplementary Table 2.** Details of the 54 metabolites between the antibiotics treated group and control group

| No. | Identify | ENR vs. CON | | | VAN vs. CON | | | PMB vs. CON | | |
| --- | --- | --- | --- | --- | --- | --- | --- | --- | --- | --- |
|  |  | ρ-value | Fold change | VIP | ρ-value | Fold change | VIP | ρ-value | Fold change | VIP |
| 1 | 5-Aminovaleric acid | <0.001 | 0.085 | 1.691 | <0.001 | 20.535 | 1.433 | 0.075 | 1.670 | 1.575 |
| 2 | Leucine | <0.001 | 0.129 | 1.679 | 0.115 | 0.266 | 0.847 | 0.579 | 1.378 | 0.546 |
| 3 | Pentadecanoic acid | <0.001 | 2.753 | 1.670 | <0.001 | 4.443 | 1.509 | 0.634 | 1.133 | 0.470 |
| 4 | 4-Hydroxyproline | <0.001 | 3.470 | 1.646 | 0.447 | 0.860 | 0.435 | 0.491 | 1.178 | 0.674 |
| 5 | Gluconic acid | <0.001 | 2.563 | 1.614 | 0.237 | 1.375 | 0.658 | 0.087 | 1.473 | 1.526 |
| 6 | 9-(Z)-Hexadecenoic acid | 0.001 | 2.019 | 1.555 | 0.128 | 1.339 | 0.822 | 0.137 | 1.422 | 1.361 |
| 7 | Tryptophan | 0.001 | 0.393 | 1.549 | 0.562 | 0.671 | 0.335 | 0.724 | 0.850 | 0.350 |
| 8 | 2,4,5-Trihydroxypentanoic acid | 0.002 | 1.988 | 1.504 | 0.012 | 0.257 | 1.207 | 0.537 | 1.217 | 0.605 |
| 9 | Glutamic acid | 0.003 | 0.203 | 1.490 | 0.552 | 0.672 | 0.343 | 0.112 | 2.314 | 1.440 |
| 10 | 4-Aminobutyric acid | 0.004 | 3.860 | 1.467 | <0.001 | 30.537 | 1.481 | 0.488 | 1.170 | 0.677 |
| 11 | Hexadecanoic acid | 0.005 | 1.500 | 1.434 | 0.011 | 1.521 | 1.213 | 0.683 | 1.071 | 0.404 |
| 12 | Tetradecanoic acid | 0.006 | 1.707 | 1.427 | 0.001 | 2.417 | 1.397 | 0.467 | 1.217 | 0.709 |
| 13 | Glycolic acid | 0.008 | 2.149 | 1.396 | 0.015 | 2.988 | 1.181 | 0.276 | 1.334 | 1.036 |
| 14 | Erythronic acid | 0.009 | 1.891 | 1.381 | 0.617 | 1.334 | 0.289 | 0.042 | 1.767 | 1.742 |
| 15 | Thymine | 0.009 | 1.977 | 1.380 | <0.001 | 4.771 | 1.484 | 0.395 | 1.270 | 0.823 |
| 16 | myo-Inositol | 0.010 | 3.431 | 1.372 | 0.645 | 0.745 | 0.267 | 0.084 | 2.088 | 1.538 |
| 17 | Methionine | 0.011 | 0.190 | 1.363 | 0.974 | 0.977 | 0.019 | 0.367 | 2.142 | 0.870 |
| 18 | 2-Hydroxyglutaric acid | 0.011 | 7.501 | 1.358 | 0.019 | 5.951 | 1.150 | 0.252 | 1.859 | 1.084 |
| 19 | Isoleucine | 0.012 | 0.333 | 1.346 | 0.937 | 1.053 | 0.046 | 0.266 | 2.600 | 1.056 |
| 21 | Phenylalanine | 0.014 | 0.425 | 1.331 | 0.923 | 1.050 | 0.056 | 0.221 | 1.500 | 1.152 |
| 22 | Ethanolamine | 0.017 | 2.199 | 1.305 | 0.001 | 9.072 | 1.424 | 0.707 | 1.135 | 0.372 |
| 23 | Uric acid | 0.021 | 0.492 | 1.278 | 0.375 | 0.374 | 0.504 | 0.494 | 0.894 | 0.669 |
| 24 | myo-Inositol-1-phosphate | 0.021 | 2.786 | 1.272 | 0.006 | 5.902 | 1.269 | 0.076 | 2.360 | 1.571 |
| 25 | Glycerol | 0.026 | 1.593 | 1.244 | <0.001 | 11.875 | 1.491 | 0.310 | 1.255 | 0.972 |
| 26 | Ribitol | 0.027 | 2.034 | 1.237 | 0.323 | 1.534 | 0.558 | 0.135 | 1.659 | 1.367 |
| 27 | Galacturonic acid | 0.030 | 1.845 | 1.219 | <0.001 | 10.268 | 1.472 | 0.124 | 1.491 | 1.401 |
| 28 | beta-Alanine | 0.031 | 1.547 | 1.217 | <0.001 | 3.266 | 1.445 | 0.947 | 1.031 | 0.066 |
| 29 | Sorbitol | 0.033 | 2.624 | 1.205 | 0.048 | 2.335 | 0.985 | 0.239 | 1.670 | 1.113 |
| 30 | Lactic acid | 0.037 | 1.502 | 1.186 | <0.001 | 9.167 | 1.464 | 0.103 | 1.327 | 1.469 |
| 31 | Pantothenic acid | 0.041 | 1.719 | 1.168 | 0.002 | 2.859 | 1.352 | 0.356 | 1.430 | 0.889 |
| 32 | Threitol | 0.041 | 2.509 | 1.167 | 0.438 | 0.769 | 0.443 | 0.215 | 1.848 | 1.165 |
| 33 | Gentiobiose | 0.041 | 1.583 | 1.167 | 0.001 | 0.340 | 1.401 | 0.134 | 1.316 | 1.372 |
| 34 | Phosphoric acid | 0.049 | 1.749 | 1.137 | <0.001 | 9.970 | 1.455 | 0.099 | 1.869 | 1.483 |
| 35 | Serine | 0.203 | 0.747 | 0.789 | <0.001 | 17.559 | 1.493 | 0.509 | 1.210 | 0.647 |
| 36 | Glutaric acid | 0.412 | 1.195 | 0.525 | <0.001 | 4.433 | 1.488 | 0.021 | 1.833 | 1.896 |
| 37 | Adenine | 0.133 | 1.563 | 0.912 | <0.001 | 66.383 | 1.479 | 0.236 | 1.314 | 1.119 |
| 38 | Uracil | 0.265 | 1.312 | 0.700 | <0.001 | 11.711 | 1.468 | 0.696 | 1.128 | 0.387 |
| 39 | Hypoxanthine | 0.329 | 1.252 | 0.618 | 0.001 | 4.933 | 1.423 | 0.518 | 1.287 | 0.634 |
| 40 | Threonine | 0.714 | 1.099 | 0.238 | 0.001 | 8.386 | 1.381 | 0.319 | 1.389 | 0.956 |
| 41 | Threonic acid | 0.051 | 1.373 | 1.130 | 0.001 | 0.382 | 1.380 | 0.100 | 1.617 | 1.480 |
| 42 | 4-Hydroxy-3-methoxybenzoic acid | 0.056 | 1.196 | 1.111 | 0.002 | 0.237 | 1.359 | 0.702 | 1.069 | 0.379 |
| 43 | Lysine | 0.483 | 0.813 | 0.451 | 0.002 | 9.422 | 1.358 | 0.838 | 1.092 | 0.203 |
| 44 | Homoserine | 0.876 | 0.951 | 0.102 | 0.002 | 3.545 | 1.351 | 0.673 | 1.199 | 0.418 |
| 45 | Fucose | 0.354 | 0.748 | 0.589 | 0.003 | 6.896 | 1.321 | 0.214 | 1.572 | 1.166 |
| 46 | Ornithine | 0.407 | 0.748 | 0.530 | 0.005 | 6.104 | 1.293 | 0.492 | 1.322 | 0.672 |
| 47 | Alanine | 0.227 | 1.222 | 0.752 | 0.005 | 3.300 | 1.282 | 0.300 | 1.224 | 0.991 |
| 48 | Arabitol | 0.064 | 1.999 | 1.083 | 0.010 | 4.917 | 1.226 | 0.341 | 1.410 | 0.916 |
| 49 | Glycerol-3-phosphate | 0.054 | 2.140 | 1.116 | 0.011 | 3.124 | 1.213 | 0.049 | 2.701 | 1.698 |
| 50 | trans-Ferulic acid | 0.690 | 0.869 | 0.259 | 0.016 | 6.552 | 1.169 | 0.814 | 0.923 | 0.234 |
| 51 | Eicosanoic acid | 0.759 | 1.061 | 0.200 | 0.016 | 1.960 | 1.168 | 0.662 | 1.105 | 0.433 |
| 52 | Nicotinic acid | 0.122 | 1.562 | 0.935 | 0.022 | 1.828 | 1.131 | 0.267 | 1.423 | 1.054 |
| 53 | Aspartic acid | 0.056 | 0.295 | 1.110 | 0.024 | 0.284 | 1.123 | 0.118 | 2.453 | 1.419 |
| 54 | Orotic acid | 0.145 | 0.217 | 0.889 | 0.024 | 0.097 | 1.121 | 0.629 | 0.781 | 0.477 |
